# Facile Preparation of Nano-Bi_2_MoO_6_/Diatomite Composite for Enhancing Photocatalytic Performance under Visible Light Irradiation

**DOI:** 10.3390/ma11020267

**Published:** 2018-02-09

**Authors:** Lu Cai, Jiuyan Gong, Jianshe Liu, Hailong Zhang, Wendong Song, Lili Ji

**Affiliations:** 1 College of Environmental and Science Technology, Donghua University, Shanghai 201620, China; lucai89@126.com; 2 College of Petrochemical and Energy Engineering, Zhejiang Ocean University, Zhoushan 316022, China; gongjiuyan@126.com (J.G.); swd60@163.com (W.S.); 3 Institute of Innovation & Application, Zhejiang Ocean University, Zhoushan 316022, China; jll-gb@163.com

**Keywords:** composite, photocatalyst, Bi_2_MoO_6_, diatomite/diatomaceous earth

## Abstract

In this work, a new nano-Bi_2_MoO_6_/diatomite composite photocatalyst was successfully synthesized by a facile solvothermal method. Scanning electron microscopy (SEM), Fourier transform infrared spectroscopy (FTIR), X-ray diffraction (XRD), and UV-vis diffuse reflection spectroscopy (DRS) were employed to investigate the morphology, crystal structure, and optical properties. It was shown that nanometer-scaled Bi_2_MoO_6_ crystals were well-deposited on the surface of Bi_2_MoO_6_/diatomite. The photocatalytic activity of the obtained samples was evaluated by the degradation of rhodamine B (RhB) under the visible light (λ > 420 nm) irradiation. Moreover, trapping experiments were performed to investigate the possible photocatalytic reaction mechanism. The results showed that the nano-Bi_2_MoO_6_/diatomite composite with the mass ratio of Bi_2_MoO_6_ to diatomaceous earth of 70% exhibited the highest activity, and the RhB degradation efficiency reached 97.6% within 60 min. The main active species were revealed to be h^+^ and•O^2−^. As a photocatalytic reactor, its recycling performance showed a good stability and reusability. This new composite photocatalyst material holds great promise in the engineering field for the environmental remediation.

## 1. Introduction

Photocatalysis with semiconductor catalysts is a promising method for removing organic compounds from water and an effective solution for the current energy and environmental crises. In recent decades, many semiconductor photocatalysts have been developed to tackle the increasing and serious water pollution [1]. TiO_2_ as a photocatalyst due to its chemical stability, low cost, non-toxicity, etc. has been studied in recent decades [2]. However, there are two main disadvantages for TiO_2_ to limit its application. First, TiO_2_ has the band gap of about 3.2 eV, which means that titanium dioxide can only be used in the case of the irradiation of ultraviolet excitation, and the ultraviolet spectrum is only 4% of the total solar light, which helps explain why the catalytic efficiency of titanium dioxide is so low [3]. Second, photoinduced electrons and holes can easily recombine on the surface of TiO_2_. This can lead to its low quantum efficiency, and furthermore lower its catalytic efficiency [4].

Bi-based photocatalysts have attracted great interests because of their visible-light-driven photodecomposition of organic contaminants [5], such as BiVO_4_ [6], BiOX (X = Br, Cl) [7,8,9], Bi_2_WO_6_ [10], Bi_2_Fe_4_O_9_ [11], and Bi_2_O_2_CO_3_ [12]. Among them, Bi_2_MoO_6_ is considered to be an emergent attractive photocatalyst [13]. As a typical aurivillius oxide, Bi_2_MoO_6_ is a promising photocatalyst under visible light irradiation, and, as an n-type semiconductor, Bi_2_MoO_6_ is arousing many more researchers’ attention. It is composed of perovskite layers (A_m−1_B_m−3_O_3m+1_) and bismuth oxide layers (Bi_2_O_2_^2+^); these layers can arrange alternatively and possess excellent photocatalytic activity under visible light irradiation. As we know that more than 43% of the solar radiance onto the Earth’s surface is visible light, compared to titanium dioxide photocatalyst, its catalytic efficiency will be much higher. To further improve photocatalytic performance of Bi_2_MoO_6_, many composite superstructures have been proposed and synthesized, for instance, TiO_2_/Bi_2_MoO_6_ [14], Bi_2_MoO_6_/MoO_3_ [15], g-C_3_N_4_/Bi_2_MoO_6_ [16], Bi_2_MoO_6_-RGO [17] and other photocatalysts have shown a high catalytic activity and light stability. However, these materials are not only high cost and difficult to prepare, but also can accelerate the agglomerate of Bi_2_MoO_6_. Some research has focused on finding hierarchical porous materials to be carrier for Bi_2_MoO_6_ [18]. Only nanometer-scaled TiO_2_ has been reported to coat on the surface of diatomite via a simple process for the production of efficient and hierarchically structured catalysts [19]. Diatomite is one of the most readily available supports due to its inherently hierarchical porosity [20,21].

Recently, diatomite, i.e., diatomaceous earth, has gained considerable attention due to its large specific surface area, special aperture structure, low cost and high chemical stability [22]. The main component of diatomite is SiO_2_. Diatomite has been used extensively as filtration media, adsorbents, ideal photocatalytic carriers and it has many silicon hydroxyl groups on its surface [23,24], which can be benefited to provide more OH sites for the adsorption of organic pollutants. However, there is little research and limited reports on diatomaceous earth. Some composites, such as TiO_2_/diatomaceous earth [24], can render significantly enhanced photocatalytic activity. This enhancement can be greatly attributed to large surface areas of synthetic composites. These large surface areas can provide more catalytic active site and enhance the photocatalytic efficiency.

In this study, we report the facile fabrication of nano-Bi_2_MoO_6_/diatomite composite by simply depositing nanometer-scaled Bi_2_MoO_6_ onto the surface of diatomite. The effect of nano-Bi_2_MoO_6_/diatomite composite on photocatalytic activity was investigated by decomposing rhodamine B (RbB) dye under visible-light irradiation. The possible photocatalytic mechanism of this composite was also discussed. The main objective of this study is to better elucidate the adsorption and photodegradation of RhB fractions by nano-Bi_2_MoO_6_/diatomites, as well as the characteristics of nano-Bi_2_MoO_6_/diatomite composites.

## 2. Materials and Methods

### 2.1. Materials

Diatomaceous earth was purchased from Henan Qikang water treatment materials Co., Ltd, Zhengzhou, Henan, China. Bi(NO_3_)·5H_2_O, Na_2_MoO_4_·H_2_O, glycol, ethanol (>99.7%) and hydrochloric acid were purchased from Sinopharm Chemical Reagent Co., Ltd, Shanghai, China. All chemicals were analytical grade and used as received without further purification. 

### 2.2. Purification of Diatomaceous Earth

A 20% HCl solution was employed to clean diatomaceous earth samples to remove the impurities, such as Fe, Al and some organic matter. The hydrochloric acid solution with 2.5:1 (*v*/*w*) liquid-to-solid ratio was prepared to mix with diatomite. The solution was stirred for 4 h and then the diatomite was washed with distilled water to neutral. Finally, the diatomite material was filtered, dried and calcined at 600 °C for 2 h.

### 2.3. Preparation of Bi_2_MoO_6_/Diatomaceous Earth Composites

The solvothermal technique was employed to synthesize Bi_2_MoO_6_/diatomaceous earth composite. First, 0.363 g Bi(NO_3_)_3_·5H_2_O and 0.0907 g Na_2_MoO_4_·2H_2_O each was dissolved in two different 7 mL ethylene glycol solutions under magnetic stirring. Then, 0.098 g diatomaceous earth was dissolved in 20 mL ethanol. The three solutions were mixed together and a magnetic stirrer was used to stir the mixed solution for 30 min. The solution was then transferred to a 50 mL Teflon-lined stainless steel autoclave, which was heated to 160 °C and then maintained at this temperature for 12 h. After cooling down to room temperature, the obtained composite was washed by filtration with ethanol and deionized water three times, respectively. Finally, the composite was centrifuged for 10 min at 8000 rpm, and then the composite was dried at 60 °C for 12 h in oven. The samples were marked as following, according to the mass ratio of Bi_2_MoO_6_ to diatomaceous earth: 0%, 50%, 60%, 70%, 80%, and 100%. The synthetic composite samples were named as diatomaceous earth, B/D-50%, B/D-60%, B/D-70%, B/D-80%, and Bi_2_MoO_6_, respectively.

### 2.4. Characterization

The phase structures of photocatalysts were investigated by X-ray powder diffraction (XRD) analysis at room temperature on an XRD powder diffraction instrument (D8 ADVANCE Da Vinci, BRUKER AXS GMBH, Karlsruhe, Germany) with monochromatized Cu Ka radiation (=0.15406 nm) at a setting of 40 kV and 40 mA. The scanning rate and the range were 0.02 (2θ)/s, and from 10 to 70, respectively. Fourier-transform infrared (FTIR) spectra were obtained using a Bruker spectrometer (Nicolet 6700, Thermo Fisher Scientific, Waltham, MA, USA) with KBr as a diluting agent and operated in the frequency range of 4000–400 cm^−1^. The morphology and the microstructure of the photocatalysts were studied by field emission scanning electron microscopy (SEM) (Hitachi-4800, Hitachi Co., Ltd., Tokyo, Japan, accelerating voltage = 15 KV). The UV-vis diffuse reflectance spectra of the samples were determined on a Shimadzu UV 2600 spectrophotometer (Shimadzu Co., Ltd., Kyoto, Japan) using BaSO_4_ as the reference material.

### 2.5. Photodegradation Experiments

To investigate the photocatalytic activity of the Bi_2_MoO_6_/diatomite composite, RhB dye was used as the organic contaminant. Fifteen milligrams of the Bi_2_MoO_6_/diatomite composite were added to 40 mL of RhB (4 mg/L) dye solution. The suspension was stirred by a magnetic stirrer for 1 h to establish an adsorption/desorption equilibrium in the dark. Then, at 10 min intervals, 2 mL of the suspension was taken to centrifuged (8000 rpm 10 min) for removing photocatalyst powder under the 300 W Xenon lamp light irradiation with a cutoff filter (l > 400 nm), and the distance between the xenon lamp and reaction cell is 50 mm. The temperature of the reaction system was kept at 25 °C by cycling water. The UV-2600 spectrophotometer was used to measure changes of the concentration of RhB solution with the wavelength of 554 nm.

The stability test was performed by the degradation of RhB solution (40 mL, 4 mg/L) over B/D-70% for four successive runs. After each cycle, the catalyst was separated and collected by a simple precipitation procedure, then was washed thoroughly and dried. After that, it was added into the fresh RhB solution (40 mL, 4 mg/L) to initiate the reaction.

## 3. Results and Discussion

**Figure 1:**
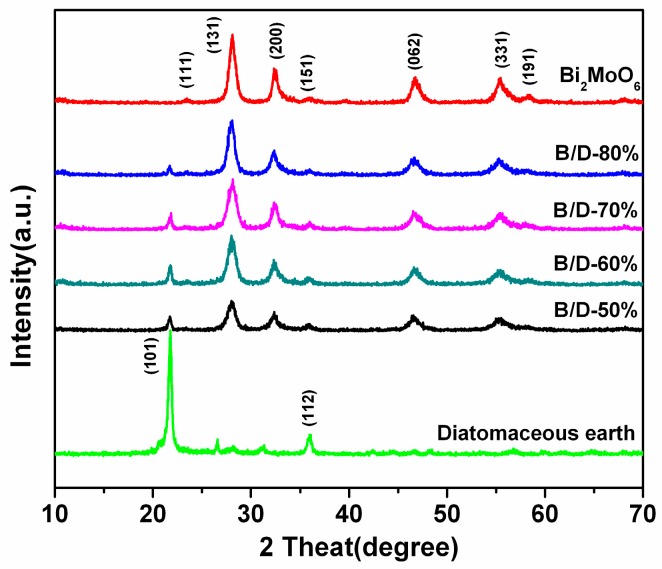
XRD patterns of Bi_2_MoO_6_, B/D-50%, B/D-60%, B/D-70%, B/D-80%, and diatomite.

The crystalline structure and the purity of the as-prepared photocatalysts are characterized by powder X-ray diffraction (XRD). Figure 1 presents the XRD patterns of pure Bi_2_MoO_6_ and nano-Bi_2_MoO_6_/diatomite with different diatomaceous earth contents. Diffraction peaks at about 2θ = 23.3, 28.1, 32.3, 35.8, 46.7, 55.3 and 58.2 appear in the spectra, corresponding to (111), (131), (200), (151), (062), (331) and (191) crystal planes of orthorhombic phase Bi_2_MoO_6_ (JCPDS Card No. 76-2388), respectively [11]. In addition, the diffraction peaks of diatomaceous earth mainly locate in the range of 20–40, and additional diffraction peaks with 2θ values of 21.7 and 35.9 can be assigned perfectly to (101) and (112) crystal faces of SiO_2_ (JCPDS Card No. 82-0512), respectively. It illustrates that the main component of diatomite is SiO_2_ crystal. Obviously, XRD pattern of Bi_2_MoO_6_/diatomaceous earth can be indexed as the mixture of Bi_2_MoO_6_ crystalline and diatomaceous earth. Therefore, the hierarchically structured composite is composed of nano feature Bi_2_MoO_6_ crystal and diatomaceous earth.

**Figure 2:**
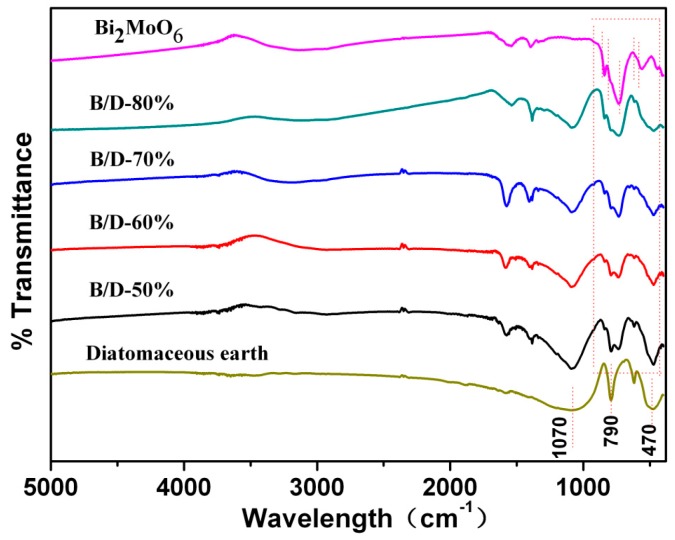
FTIR spectra of Bi_2_MoO_6_, B/D-50%, B/D-60%, B/D-70%, B/D-80%, and diatomite.

The FTIR spectra of Bi_2_MoO_6_, diatomaceous earth and their composites are shown in Figure 2. It can be observed that there are peaks at 1070 cm^−1^ (antisymmetric vibration mode of Si-O-Si), 790 and 470 cm^−1^ (the vibration of the Si-O bond) for the pure diatomaceous earth [12]. The Bi_2_MoO_6_ only exhibits the absorption peaks around the range of 400–900 cm^−1^. The peaks at around 845 and 798 cm^−1^ can be distribution as the antisymmetric vibration mode and symmetric stretching mode of MoO_6_^6+^, respectively. The band at 734 cm^−1^ is attributed to the asymmetric stretching mode of MoO_6_^6+^ involving vibrations of the equatorial oxygen atoms and the bands at 605 cm^−1^, 568 cm^−1^ correspond to the bending vibration of MoO_6_^6+^. Compared with the spectrum curves in Figure 2, all the nano-Bi_2_MoO_6_/diatomite composites present peaks of pure Bi_2_MoO_6_ and diatomaceous earth, which implies that nanojunctions of the Bi_2_MoO_6_/diatomaceous earth are successfully formed.

**Figure 3:**
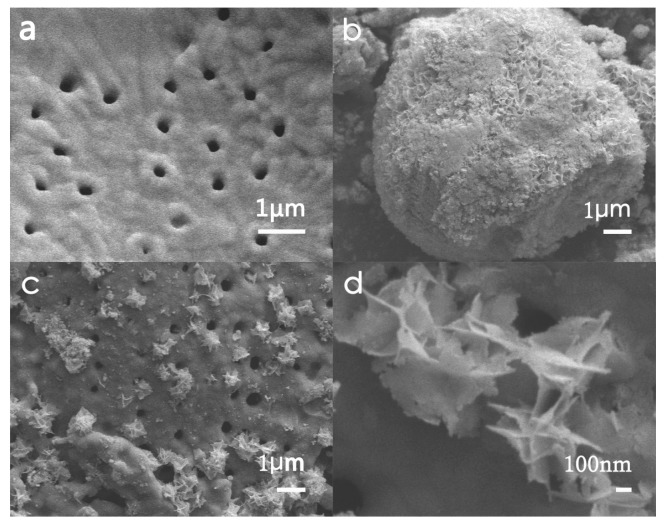
SEM images of: diatomite (**a**); Bi_2_MoO_6_ (**b**); and B/D-70% with different magnifications (**c**,**d**).

Some typical SEM photographs of diatomaceous earth, Bi_2_MoO_6_, and nano-Bi_2_MoO_6_/diatomite composites-B/D-70% are shown in Figure 3. The morphology of the diatomaceous earth sample appears disc-like, and the diameter of the average shell is measured as about 28 μm. Local magnification shows that diatomite has a highly developed micro-porous structure, the shell surface is clean and smooth after purification with a 20% HCl solution, and there are almost no impurities or debris attached, as shown in Figure 3a. Figure 3b shows that pure Bi_2_MoO_6_ crystal normally appeared in spherical form, and the agglomerates of Bi_2_MoO_6_ have an average size of 3–8 μm in diameter. Figure 3c illustrates some details of morphology of nano-Bi_2_MoO_6_/diatomite composites, and the distribution of nanometer-scaled Bi_2_MoO_6_ on the surface of diatomite is copious and homogeneous. In Figure 3c,d, it can be clearly seen that synthesized Bi_2_MoO_6_ crystals loaded on the surface of diatomite appear about 1 μm nest-like structures with some nano-featured flakes and a large amount of Bi_2_MoO_6_ particles are deposited on the surface and pores of diatomaceous earth (Figure 3c,d), indicating that Bi_2_MoO_6_ particles adhere well to diatomaceous earth to form a good composite photocatalyst. On the other hand, the Bi_2_MoO_6_ crystals synthesized without the presence of diatomaceous earth appear to be microspheres, as shown in Figure 3b. In Figure 3b, we can clearly see some nano-flake features on the surface of the microspheres of Bi_2_MoO_6_. However, this aggregation form of Bi_2_MoO_6_ can only make its photocatalysis less efficient.

**Figure 4:**
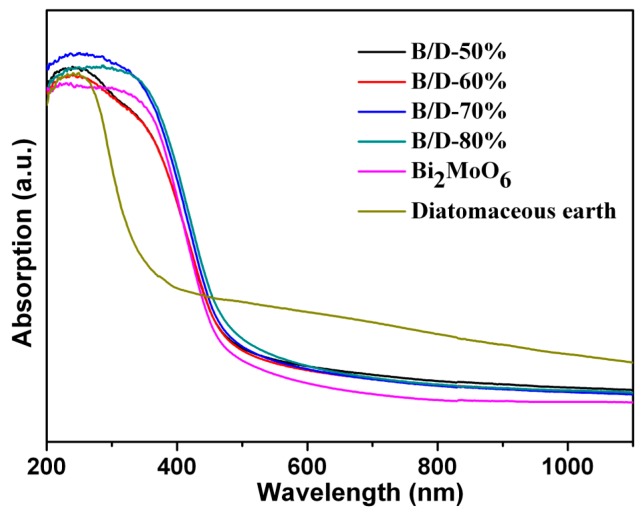
UV-vis DRS spectra of Bi_2_MoO_6_, B/D-50%, B/D-60%, B/D-70%, B/D-80%, and diatomite.

Optical absorption properties of a photocatalyst are relevant to its electronic structure features and are also recognized as key factors in determining its photocatalytic activity. To study the optical response of as-prepared samples, UV-vis absorption spectra were measured, as shown in Figure 4, the nano-Bi_2_MoO_6_/diatomite composite photocatalysts with different amounts of Bi_2_MoO_6_ exhibited slightly higher absorption than both individual pure Bi_2_MoO_6_ and pure diatomaceous earth in the UV and visible light region. The absorption increases slightly as the amount of Bi_2_MoO_6_ increases in the composites. As a carrier and support material, diatomaceous earth within the composite does not seem to provide much contribution to the optical absorption property of the composites. Pure Bi_2_MoO_6_ by itself exhibits a wavelength absorption edge at approximately 470 nm [25]. Diatomaceous earth has an intense absorption band with a steep edge at about 390 nm. These facts indicate that Bi_2_MoO_6_/diatomaceous earth composites have a relative wide region of visible light photo-response, and therefore can be expected to act as excellent visible-light-driven photocatalysts.

**Figure 5:**
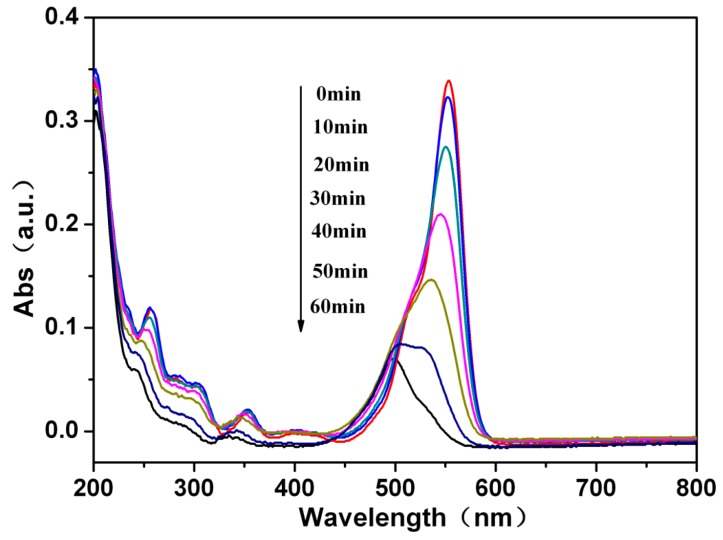
Absorption spectra of the RhB solution after different irradiation time in the presence of photocatalyst composite B/D-70%.

The visible light photocatalytic activity of the as-prepared samples has been evaluated by means of the degradation of RhB in aqueous solution. Figure 5 shows the temporal evolution of absorption spectra of RhB dye (4 mg/L, initial concentration) and photodegradation by nano-Bi_2_MoO_6_/diatomite composite photocatalysts within the time interval of 10 min. RhB has a major absorption band at around 554 nm. The peak intensity of RhB at around 554 nm decreases gradually, which indicates the RhB dye is degraded or decomposed. Consequently, RhB dye is completely degraded by nano-Bi_2_MoO_6_/diatomite composite photocatalysts with increasing illumination time. The introduction of diatomite into Bi_2_MoO_6_ apparently induces a synergistic effect on RhB degradation.

**Figure 6:**
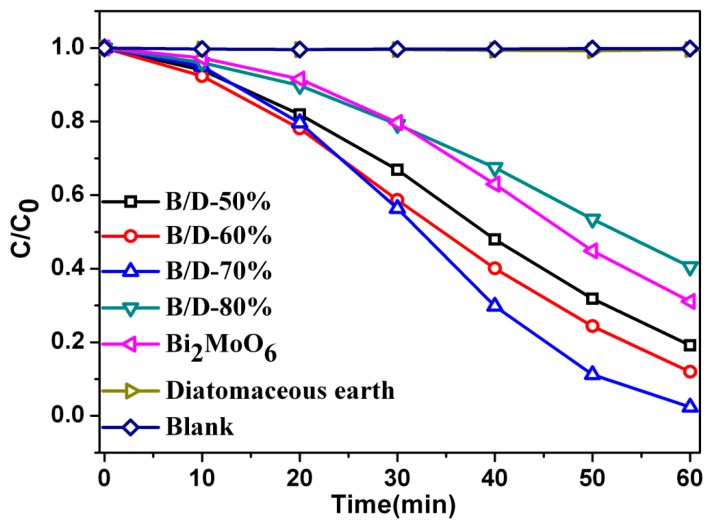
Degradation efficiency of RhB for Bi_2_MoO_6_, B/D-50%, B/D-60%, B/D-70%, B/D-80%, and diatomite.

Figure 6 shows results of the photocatalytic efficiency of degradation of RhB solution by different photocatalysts. For the controlled experiment, RhB solutions without photocatalyst (blank test) and with diatomaceous earth are also measured under the same conditions for as-prepared samples with Bi_2_MoO_6_, as illustrated in Figure 6. The degradation of RhB is extremely slow without photocatalyst or with diatomaceous earth. By using only the Bi_2_MoO_6_ as photocatalyst, the photodegradation efficiency of RhB can approach 68.9% after 60 min reaction. This indicates that as-prepared samples containing higher mass ratio of Bi_2_MoO_6_ to diatomite exhibits higher photocatalytic activity than the sample diatomaceous earth by itself. It is obvious that the degradation efficiency increases gradually as the content of Bi_2_MoO_6_ increases in the composite photocatalysts from B/D-50% to B/D-70% and degradation efficiency of the B/D-70% reaches the maximum about 97.6% under the visible light irradiation for 60 min. The degradation efficiency of B/D-50% and B/D-60% is 80.8% and 88%, respectively. This result may suggest that diatomaceous earth provides more photocatalytic activities due to its large surface area and special aperture structure [26]. However, for the sample B/D-80%, its degradation efficiency of RhB decreases to 58% in comparison with the B/D-70%. More Bi_2_MoO_6_ available may become aggregates on the surface of Bi_2_MoO_6_/diatomite, which leads to less effective adsorption sites and less photolytic active sites. As a result, the nano-Bi_2_MoO_6_/diatomite composites might have the ability to enhance the photocatalytic activity under visible-light irradiation, and the B/D-70% is the best mass ratio of Bi_2_MoO_6_ to diatomaceous earth for the degradation of RhB solution.

To better compare the photocatalytic efficiency of the as-prepared samples, the kinetic process of RhB degradation is also investigated through the photodegradation rate constant (Kapp, min^−1^), which follows the apparent first-order kinetic equation [27]:

(1) $$\ln\left(\frac{C_{0}}{C_{t}}\right)=K_{app}t$$

where *C*_0_ is the initial concentration of RhB, and *C*_t_ is the concentration of RhB under light irradiation at the time, t.

**Figure 7:**
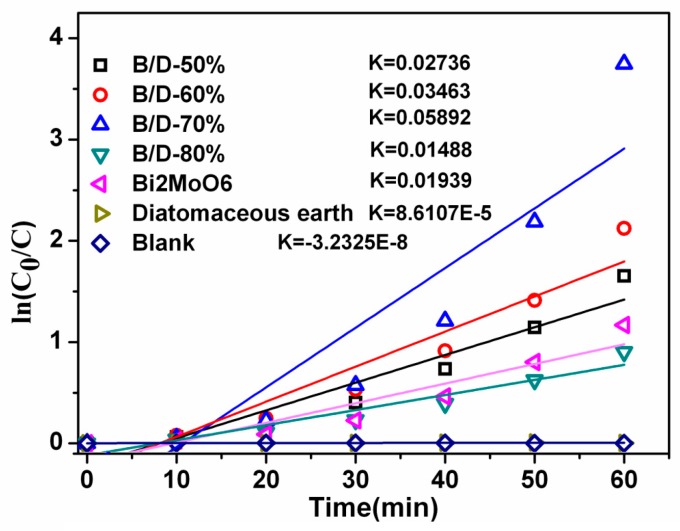
Kinetic process of RhB degradation for Bi_2_MoO_6_, diatomite, B/D-50%, B/D-60%, B/D-70%, and B/D-80%.

Figure 7 shows that the K_app_ values increase gradually from B/D-50% to B/D-70% and the K_app_ value of the B/D-70% reaches the maximum about 0.05892 min^−1^, which demonstrates that the photocatalytic efficiency of the degradation of RhB for B/D-70% composite is the highest. The K_app_ value for the sample B/D-80% decreases in comparison with the B/D-70%, which coincides with its degradation efficiency of RhB. The kinetic study indicates that the degradation of RhB solution by as-prepared photocatalyst composites follows the pseudo-first-order kinetics [28,29], probably due to its hierarchical architecture with numerous nanopores of the composites. However, especially for B/D 70%, its reaction does not seem to follow the pseudo-first-order kinetics well, this is probably due to the strong adsorption from diatomaceous earth surface. Because diatomite itself has strong adsorbability, the degradation reaction does not seem to take place in the first 10 min. This lag may cause a deviation from the reaction point. When more the dye molecules were degraded, more active groups on the surface of catalyst would be available and increase, leading to an increase of photocatalytic efficiency of B/D 70%. The result obtained in this work is similar to the previous studies of other semiconductors such as Bi_2_MoO_6_/TiO_2_ with K = 0.03505 min^−1^ [4]; Ag_3_VO_4_/Bi_2_MoO_6_, 0.0201 min^−1^ [30]; Ag_2_O/TaON, 0.0393 min^−1^ [31]; andFe_2_O_3_/Bi_2_WO_6_, 0.08586 min^−1^ [29].

**Figure 8:**
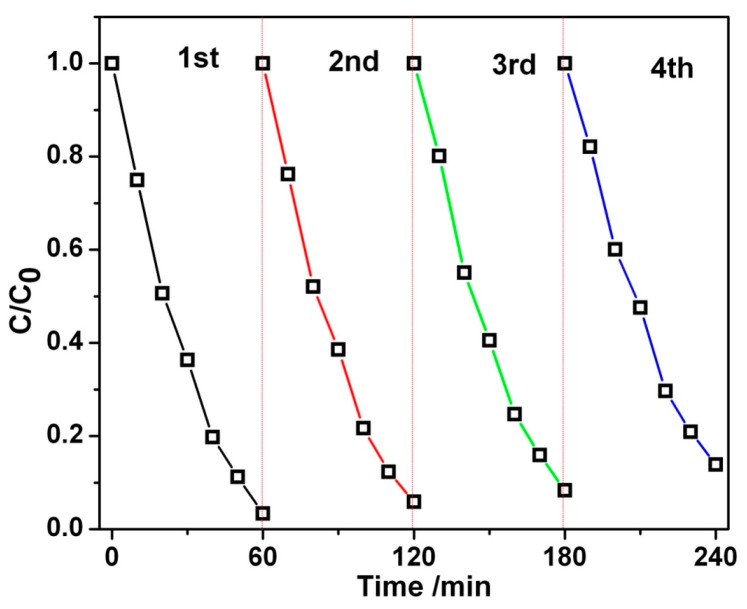
Photodegradation of RhB solution with a composite photocatalyst B/D-70% in different testing cycles.

As a photocatalytic reactor, its recycling performance has been evaluated. As shown in Figure 8, the photocatalytic degradation of RhB was monitored and measured for four testing cycles (each cycle for 60 min). For the first run, 96.6% of RhB was degraded after 60 min under visible light irradiation; subsequently, the photodegradation efficiency was 94.1%, 91.6%, and 86.1% for the second, third, and fourth run, respectively. These results demonstrated that nano-Bi_2_MoO_6_/diatomite composites had good stability and reusability in degrading RhB solution.

**Figure 9:**
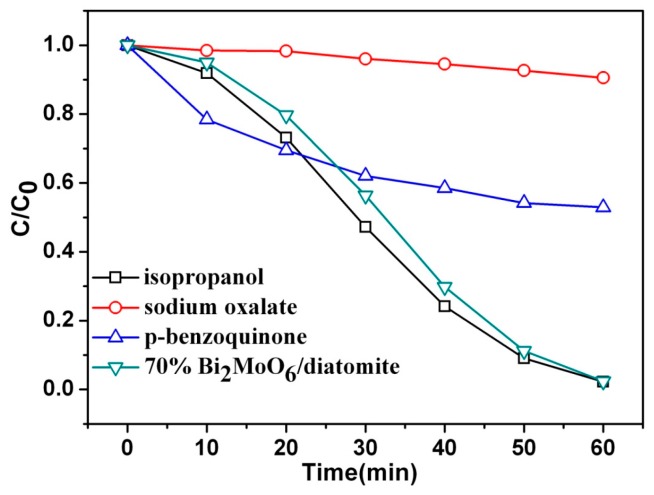
Effect of different kinds of trapping agents on the degradation of RhB solution by visible light irradiation.

During the photocatalytic oxidation process, the photocatalyst forms photogenerated electrons and holes under the visible light radiation, and pollutants are initially degraded. Electron–holes react with H_2_O and O_2_ in the solution to form active species such as radicals like •OH and •O^2−^ to further degrade the pollutants. To speculate the possible reaction mechanism for nano-Bi_2_MoO_6_/diatomite composite photocatalysts to degrade RhB, the experiment for capturing active species is designed. Wherein, isopropanol is an •OH trapping agent; sodium oxalate is a hole trapping agent; and p-benzoquinone is an •O^2−^ trapping agent. The photocatalytic degradation efficiency of RhB after adding each of these three capture agents, such as sodium oxalate, p-benzoquinone and isopropanol, to the RhB testing solutions is shown in Figure 9 and the B/D-70% composite photocatalyst was selected for the testing experiments. As illustrated in Figure 9, the degradation efficiency of RhB decreased from 97.6% to 9.4%, 47.1% and 97.5%, respectively. The results showed that the addition of sodium oxalate and p-benzoquinone had a strong inhibitory effect on the catalytic activity of Bi_2_MoO_6_/diatomaceous earth composite photocatalyst. This may be because the hole is captured by sodium oxalate, resulting in a decrease in the number of holes and a decrease in the number of •OH active species (hole + OH^−^ → •OH) generated by the reaction of holes and •OH, and in consequence the final photocatalytic activity is reduced. The benzoquinone captures the active species •O^2−^, reducing the amount of •O^2−^, thereby reducing the photocatalytic activity of the catalyst. It may suggest that h+ and •O^2−^ play a role in the photocatalytic degradation of RhB in Bi_2_MoO_6_/diatomaceous earth composite catalyst, in which h^+^ and •O^2−^ were the main active species for the degradation of RhB.

**Figure 10:**
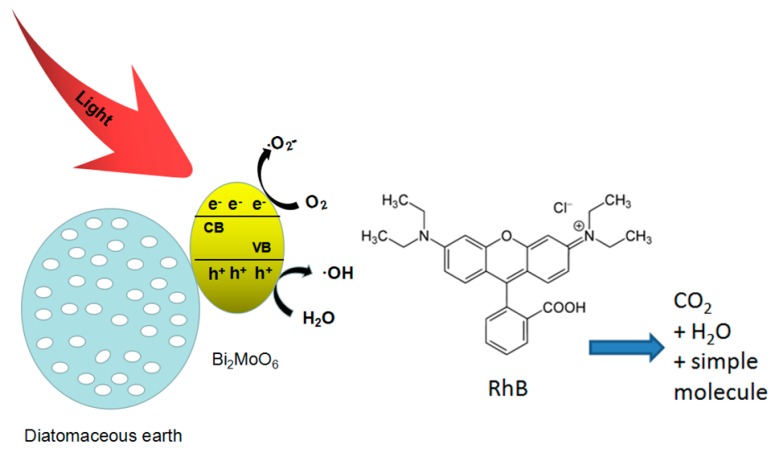
Schematic diagram of a possible photocatalytic mechanism about nano-Bi_2_MoO_6_/diatomite under visible irradiation.

Based on the results of the determination of the active species and the some related literature [32,33,34], a possible reaction mechanism is proposed to explain the enhancement of the photocatalytic activity of nano-Bi_2_MoO_6_/diatomite composites. In this system, as illustrated in Figure 10, as-prepared nano-Bi_2_MoO_6_/diatomite composites present the morphology with Bi_2_MoO_6_ nano-flakes attached homogeneously onto the surface of diatomite, rather as-prepared pure Bi_2_MoO_6_ sample (synthesized without the presence of diatomite) has developed a microspheres-like morphology, i.e., the agglomerate of Bi_2_MoO_6_. These large surface areas of the as-prepared composites can provide more catalytic active sites and enhance their photocatalytic efficiency. The whole process of the generation, transportation, and reactions of photoinduced charge carriers in the composites is shown in Figure 10. Upon the visible light (λ > 420 nm) irradiation onto these nanometer-scaled Bi_2_MoO_6_ crystals, within the nano-Bi_2_MoO_6_/diatomite composites, more surface areas available mean more electrons are excited from valence band to conduct band. With the transition of electrons, photogenerated electron–hole pairs are then formed. Photogenerated electrons react with O_2_ adsorbed on the surface of the catalyst and the composites to produce active species •O^2−^, while the separation of holes and H_2_O reaction to produce strong oxidizing active species •OH. It is noted that the diatomaceous earth within the composites has a pore structure with a large specific surface area, which is conducive to the absorption of the visible light; it may help the generation of photogenerated carriers and help provide the sufficient sites for adsorption of contaminants. Therefore, the photocatalytic performance of nano-Bi_2_MoO_6_/diatomite is enhanced eventually.

## 4. Conclusions

In summary, a novel nano-Bi_2_MoO_6_/diatomite composite photocatalyst has been successfully prepared via a simple solvothermal method. This facile and effective strategy has been demonstrated to form nest-like nano-cluster Bi_2_MoO_6_ crystals on the surface of diatomite to construct hierarchical photocatalytic reactor. This composite reactor is found clearly to enhance the degradation rate compared with pure Bi_2_MoO_6_. Under the visible light irradiation (λ > 420 nm), the optimum photocatalytic efficiency of nano-Bi_2_MoO_6_/ diatomite composite photocatalysts for the degradation of RhB is significantly higher than that of individual Bi_2_MoO_6_. Results from the trapping experiments suggest that O^2−^ and h^+^ play a role in the degradation of RhB solutions. This enhancement of photocatalytic activity of nano-Bi_2_MoO_6_/diatomite composite is contributed to the synergistic effect of visible light absorption and effective electron–hole separation at the interfaces of the composites. The nano-Bi_2_MoO_6_/diatomite photocatalysis experiment shows that combining the adsorption of the nano-Bi_2_MoO_6_/diatomite and the photochemical catalysis of nano-Bi_2_MoO_6_ can effectively degrade RhB. 

Further study is needed to fully understand this enhancement mechanism. In this work, the nano-Bi_2_MoO_6_/diatomite composite photocatalytic reactor is found to have a good stability which may suggest that it can be easily recycled in future applications. This inexpensive composite photocatalyst has potential in environmental remediation applications.
